# Acute Ekbom’s syndrome in a patient with acute urethritis

**DOI:** 10.1192/j.eurpsy.2022.1223

**Published:** 2022-09-01

**Authors:** A. Martínez Muelas, D. Paiva Pajares, M. López Isern, P. Ivanov, M. Sánchez Pérez

**Affiliations:** Hospital Sagrat Cor, Hermanas Hospitalarias, Psychiatry, Martorell (Barcelona), Spain

**Keywords:** urethritis, emergency room, Ekbom’s syndrome

## Abstract

**Introduction:**

Delirium of parasitosis was first described by Karl Ekbom in Sweden in 1938. It is a hallucinatory monothematic delirium characterized by the unwavering conviction of having the skin infested with insects or parasites. Multiple etiologist has been described such as psychiatric and neurological disorders, substance intoxication or other medical conditions. We present a case of debut of Ekbom’s syndrome in an individual recently diagnosed with acute urethritis on antibiotic treatment.

**Objectives:**

To report a case of a patient with a debut of Ekbom’s syndrome and acute urethritis.

**Methods:**

A 40-year-old man with no previous psychiatric history is admitted psychiatric emergency room accompanied by his wife for intense anxiety and isolation at home. During the examination, the patient explains a lot of fear of a series of bugs such as bees and small parasites that invade him. The onset of symptomatology coincides with a diagnosis of chlamydia urethritis and the initiation of treatment with ceftriaxone 500mg IM + Azithromycin 1g VO. Complete physical examination is performed without alterations. Toxicological, biochemistry, hormonal and vitamin study did not show any alterations.

**Results:**

Antipsychotic treatment was started with Olanzapine up to 10mg/day and supportive treatment with benzodiazepines. The patient showed rapid improvement. At discharge, he is asymptomatic from the urological and psychopathological point of view.

**Conclusions:**

Ekbom’s syndrome is a multifactorial disorder. The patient was diagnosed of an acute psychotic disorder due to another medical condition and/or treatment with antibiotics.

**Disclosure:**

No significant relationships.

